# Plasma levels of amino acids and derivatives in retinopathy of prematurity

**DOI:** 10.7150/ijms.63603

**Published:** 2021-08-27

**Authors:** Yedi Zhou, Yu Xu, Xiang Zhang, Qian Huang, Wei Tan, Yonghui Yang, Xiaori He, Shigeo Yoshida, Peiquan Zhao, Yun Li

**Affiliations:** 1 Department of Ophthalmology, The Second Xiangya Hospital, Central South University, Changsha, Hunan, China; 2 Hunan Clinical Research Center of Ophthalmic Disease, Changsha, Hunan, China; 3 Department of Ophthalmology, Xinhua Hospital Affiliated to Shanghai Jiao Tong University School of Medicine, Shanghai, China; 4 Department of Neonatology, The Second Xiangya Hospital, Central South University, Changsha, Hunan, China; 5 Department of Ophthalmology, Kurume University School of Medicine, Kurume, Fukuoka, Japan

## Abstract

**Background:** Retinopathy of prematurity (ROP) is a retinal disease that causes blindness in premature infants. This study aimed to reveal the changes in amino acids and derivatives in the plasma of ROP patients compared with premature infants without ROP.

**Methods:** Metabolomics targeting amino acids and their derivatives was conducted to assess their plasma levels in ROP patients (*n*=58) and premature infants without ROP (*n*=25), and KEGG pathway analysis was used to identify the involved pathways.

**Results:** Among the 31 assessed metabolites, the levels of 4 amino acids were significantly altered in the ROP group. Creatinine was downregulated in the plasma of the ROP patients, while the levels of citrulline, arginine, and aminoadipic acid were upregulated in the ROP group. Significant correlations were identified between the ROP stage and plasma levels of citrulline, creatinine, and aminoadipic acid. The involved pathways included biosynthesis of amino acids, arginine and proline metabolism, and arginine biosynthesis.

**Conclusion:** The plasma levels of citrulline, creatinine, arginine, and aminoadipic acid were significantly changed in ROP patients. These metabolites could be considered potential biomarkers of ROP, and their related metabolic pathways might be involved in ROP pathogenesis.

## Introduction

Retinopathy of prematurity (ROP) is a leading cause of irreversible pediatric blindness in premature infants with low birth weight 1. The hypoxia and ischemia due to the arrested development of the retinal vasculature may cause pathological neovascularization, tractional retinal detachment and blindness. This severe process threatens the visual development of newborns 2. Even though treatments like retinal photocoagulation and intravitreal therapy of anti-vascular endothelial growth factor (VEGF) agents can be timely applied for ROP, persistent sequelae and controversies still remains 3 due to the limited understanding of ROP pathogenesis and regulations.

The pathogenesis of ROP as well as its comorbidities inevitably result in profound changes in metabolism and nutrition. Particularly, previous studies have indicated the importance of amino acids in ROP pathogenesis 4, 5. Better nutrition, especially supplementation of amino acids is essential for the synthesis of growth factors like insulin-like growth factor-1 (IGF-1), which plays a crucial role in the progress of ROP 6. Hence, nutritive factor supplements might be effective methods of preventing or treating ROP 7. On the other hand, breast milk feeding, parenteral nutrition volume, and intake of vitamins may affect rates of ROP surgery requirement 8. Supplements of nutrition for newborn infants are novel strategies for restoring energy supply and may be effective in protecting the vasculature of the retinas, and some nutraceuticals may aid in preventing ROP occurrence 9. Moreover, accumulating evidence indicates that a higher intake of amino acids significantly reduces ROP but not severe ROP 4. Therefore, the role of amino acids and derivatives during the pathological process of ROP remains unknown and is worthy of further exploration.

By performing untargeted metabolomics analysis, we recently revealed the metabolomic profiles in plasma of ROP patients who required treatment, and numerous altered metabolites were recognized, including a large proportion of amino acids and derivatives 10. However, the level of metabolites cannot be accurately quantified by the screening of untargeted metabolomics. To validate the metabolite changes in plasma, in the present study, we performed quantitative metabolomics targeting amino acids and derivatives.

## Materials and Methods

### Study subjects

In total, 83 preterm newborns were enrolled in the study between November 2019 and February 2021 at the Second Xiangya Hospital of Central South University and Xinhua Hospital affiliated with Shanghai Jiao Tong University School of Medicine. Among them, 58 infants with a diagnosis of ROP who required further treatment, such as intravitreal injection of anti-VEGF agents and laser photocoagulation, followed the ICROP protocol 11. The blood draws were conducted before treatment. Another 25 preterm newborns without retinopathy were enrolled in the control group, and their plasma was obtained together with routine clinical blood draws. The exclusion criteria were as previously described 10. The protocol was approved by the Ethics Committee of the Second Xiangya Hospital of Central South University and adheres to the tenets of the Declaration of Helsinki. Informed consent was obtained from the guardians of the participants with explanations of the nature and possible consequences of the study.

### Blood sampling and preparation

Peripheral blood samples were collected in heparin anticoagulation collection tubes. The samples were centrifuged, and the plasma was isolated and kept at -80°C after quick freezing in liquid nitrogen.

The plasma samples were thawed (4 ℃), and a 50 μL aliquot was mixed with 50 μL internal standard and 450 μL cold methanol/acetonitrile. After vortexing (60 s), ultrasonication (30 min, twice), and protein precipitation (-20°C, 1 h), the mixture was centrifuged (14000 g, 4°C, 20 min). The supernatants were vacuum dried, evaporated and redissolved in 100 μL methanol/acetonitrile solvent.

### HPLC-MS/MS analysis

Targeted metabolomics was performed using a UHPLC (1290 Infinity LC, Agilent Technologies) coupled to a QTRAP (AB Sciex 5500). The mobile phase consisted of 0.08% FA in 25 mM ammonium formate and 0.1% FA in ACN. For each sample, 2 μl aliquots were automatically injected at 4°C with a column temperature of 40°C, and the flow rate was 250 μl/min.

The gradient was as follows: 0-12 min, with liquid B changing linearly from 90% to 70%; 12-18 min, from 70% to 50%; 18-25 min, from 50% to 40% and kept for 5 min, and then liquid B was increased to 90% in 30-30.1 min and kept for 30.1-37 min. In ESI positive modes, the conditions were set as follows: source temperature 500℃; ion source gas 1 (Gas1): 40 psi; ion source gas 2 (Gas2): 40 psi; curtain gas (CUR): 30 psi; and ion spray voltage floating (ISVF): 5500 V. MRM mode was adopted to detect the amino acids and derivatives.

### Data processing and statistical analysis

To extract chromatographic peak area and retention time, MultiQuant software was used. Standards were used for correction of retention time and identification of the metabolites 12.

To identify the significantly altered metabolites, statistical analyses between the two groups were conducted by the Mann-Whitney U test. Metabolites with p values<0.05 were considered statistically significant. Receiver operating characteristic (ROC) curve analysis was conducted to assess the sensitivity and specificity of the significantly altered metabolites. The area under the ROC curve (AUC) was calculated in the ROC analysis. Multivariable logistic regression was applied to calculate the AUC of the combined biomarker panel. Spearman correlation analysis was used to determine the correlations between the ROP stage and plasma levels of metabolites.

Kyoto Encyclopedia of Genes and Genomes (KEGG) pathway analysis (https://www.kegg.jp/) was performed to identify the pathways involved in the altered metabolites.

## Results

### Overall characteristics of the study subjects

Eighty-three preterm newborns were enrolled in this study, including 58 ROP infants (male/female: 34/24) and 25 control subjects without retinopathy (male/female: 13/12). Demographic features of the recruited patients are shown in Table 1. There was no significant difference in sex or body weight at the blood draw. However, gestational age at birth, birth weight, and postmenstrual age at blood draw were different between the groups. There were significant differences between the ROP and control groups in gestational age and birth weight, probably because of the intrinsic susceptibility to ROP in younger and smaller premature newborns; these findings agree with those of previous reports. In addition, the postmenstrual age at blood draw was slightly larger in the ROP group than in the control group (P=0.0409), which is due to ethical considerations. In this study, for the control group, we utilized the blood sample collected at the last blood workup before being discharged from the NICU; our rationale was that normal premature babies should not suffer an extra blood draw only to ensure that the baseline of the two groups is consistent.

**Figure 1:**
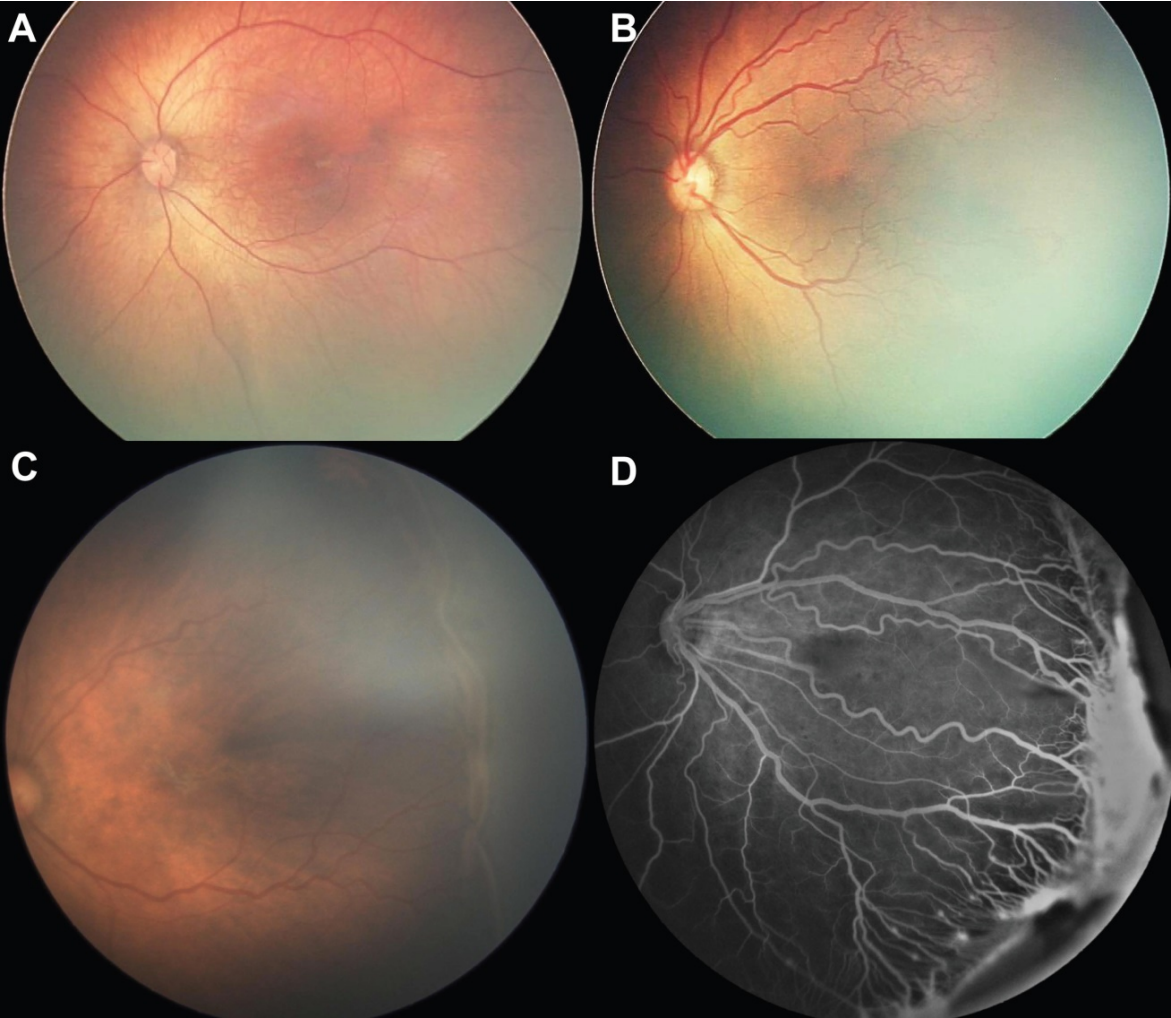
Clinical manifestation of normal premature infants and those with retinopathy of prematurity who require treatment. (A) Fundoscopy of a premature infant in the control group; (B) Fundoscopy of aggressive posterior ROP: the retina is only vascularized within Zone 1, with apparent tortuosity of retinal arteries and dilation of veins (plus disease); (C) Fundoscopy of stage 3 ROP with plus disease in posterior Zone 2; (D) Fundus fluorescein of ROP, in which prominent retinal neovascularization, plus disease and dragged disc are noted.

All the subjects in the ROP group exhibited the characteristics associated with the need for treatment; these characteristics are illustrated in Fig 1. The control group subjects (Fig. 1A) exhibited normal retinal structure and vasculature despite the premature birth. Lesions that required treatment were aggressive posterior ROP (retinal vascularization within Zone 1, plus disease defined as tortuosity of retinal arteries and dilation of veins, lacking the typical ROP ridge, Fig. 1B) and Type 1 ROP (stage 3 ROP with large-scale or plus disease, Fig. 1C). Fundus fluorescein angiography (Fig. 1D) was used to demonstrate vascular changes in ROP, i.e., prominent retinal neovascularization on the ridge, plus disease and dragged disc.

### Plasma levels of amino acids and derivatives of the participants

**Figure 2:**
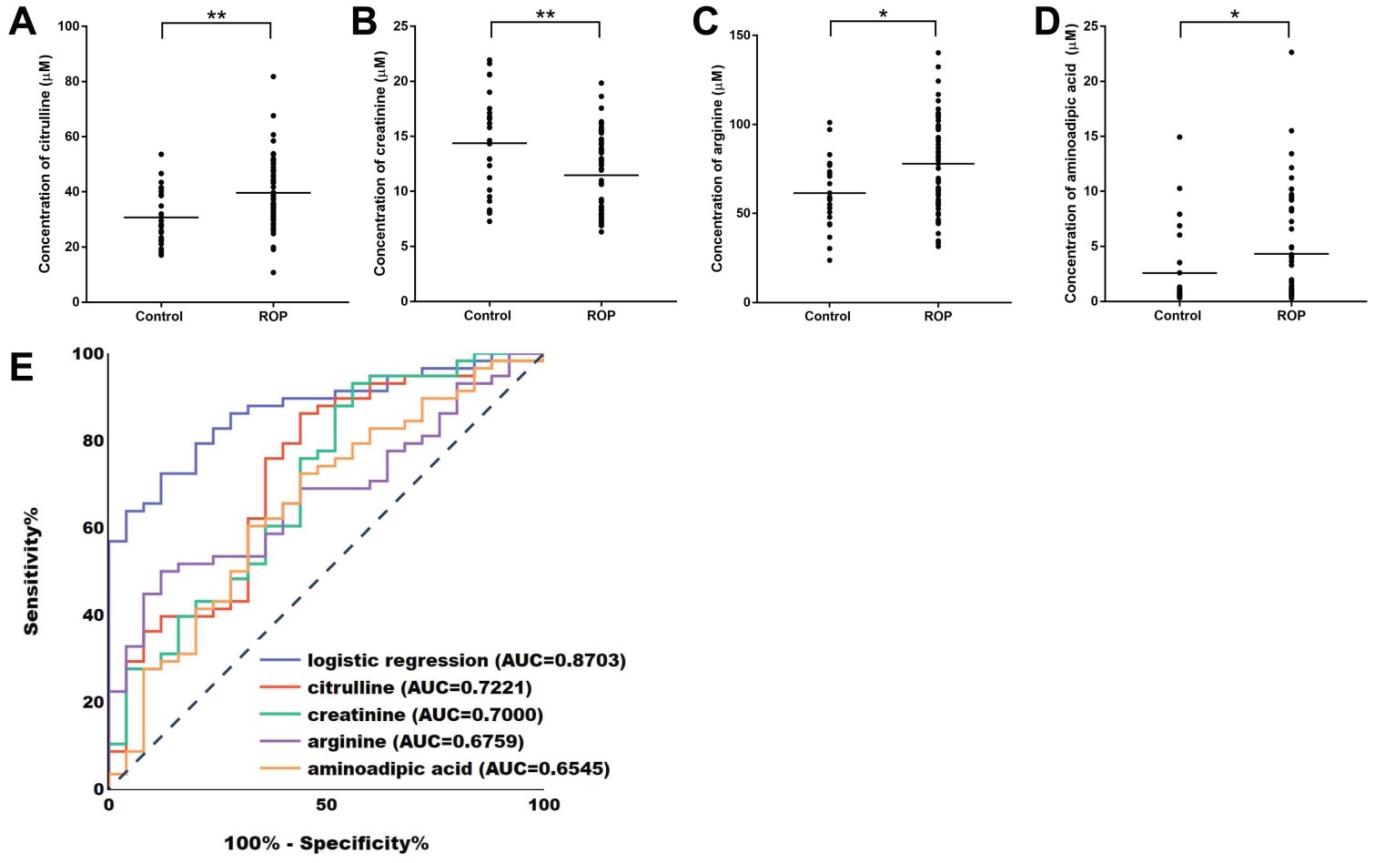
Plasma levels and ROC curves of the significantly altered metabolites. Plasma concentrations of citrulline (A), creatinine (B), arginine (C), and aminoadipic acid (D) in the control group and the ROP group. ROC curves of citrulline, creatinine, arginine, aminoadipic acid and the panel comprising the 4 metabolites by logistic regression analysis (E). ROC, receiver operating characteristic; AUC, area under the ROC curve.

To identify the plasma level of amino acids and derivatives in ROP infants, we performed a targeted metabolomics analysis of 31 metabolites (Table 2). In total, 83 samples (58 ROP and 25 controls) were assessed by the targeted metabolomics kit. The results showed that the levels of 4 metabolites were significantly different in the plasma of the ROP subjects. According to the quantification experiments, creatinine was significantly decreased (FC=0.7972, P=0.0036, Fig. 2B), while citrulline (FC=1.2912, 0.0012, Fig. 2A), arginine (FC=1.2675, P=0.0109, Fig. 2C) and aminoadipic acid (FC=1.6681, P=0.0258, Fig. 2D) were significantly upregulated in the plasma of the ROP subjects.

### Biomarker identification of the significantly altered metabolites

Next, ROC curve analyses were conducted to assess the sensitivity and specificity of the 4 altered metabolites. The AUC values for citrulline, creatinine, arginine, and aminoadipic acid were 0.7221, 0.7000, 0.6759, and 0.6545, respectively (Fig. 2E, Table 3). Moreover, multivariable logistic regression analysis demonstrated that the combination of the 4 altered metabolites could discriminate ROP infants from controls with an AUC of 0.8703 (Fig. 2E).

Furthermore, when the cutoff values were >32.04, <14.29, >61.64 and >1.135 (µmol/L), the diagnostic sensitivities/specificities of citrulline, creatinine, arginine and aminoadipic acid were 75.86/64.00, 75.86/56.00, 65.52/60.00 and 60.34/68.00 (%), respectively (Table 3).

### Correlation analysis between the ROP stage and plasma levels of the significantly altered metabolites

**Figure 3:**
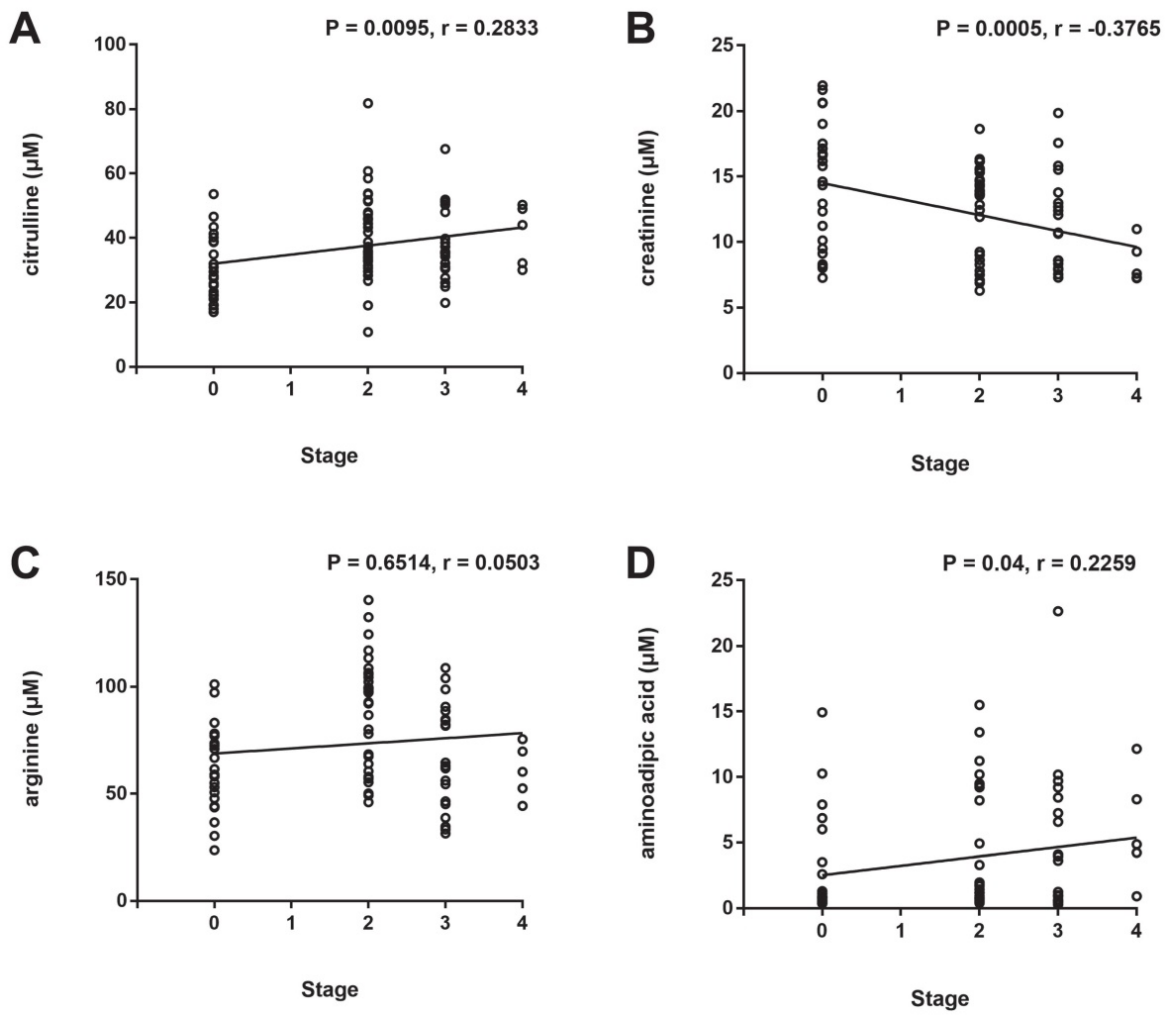
Correlation analysis between the ROP stage and plasma levels of citrulline (A), creatinine (B), arginine (C), and aminoadipic acid (D).

To assess the correlations between the ROP stage and plasma levels of the significantly altered metabolites, Spearman correlation analysis was conducted. A significant positive correlation between the ROP stage and plasma levels of citrulline (Fig. 3A, P = 0.0095, r = 0.2833) and aminoadipic acid (Fig. 3D, P = 0.04, r = 0.2259) and a significant negative correlation between the ROP stage and plasma level of creatinine (Fig. 3B, P = 0.0005, r = -0.3765) were observed. No significant correlation was found between the ROP stage and plasma level of arginine (Fig. 3C, P = 0.6514, r = 0.0503).

### KEGG pathway enrichment analysis of the significantly altered metabolites

We finally explored the enriched pathways of these 4 altered metabolites (citrulline, creatinine, arginine, and aminoadipic acid) by KEGG pathway enrichment analysis. The following enriched pathways involve at least 2 of the altered metabolites: (1) metabolic pathways; (2) biosynthesis of amino acids; (3) arginine and proline metabolism; and (4) arginine biosynthesis (Table 4).

## Discussion

Metabolomics is applied to conduct comprehensive analyses of small molecules in a biological system, and MS is a useful method for revealing the wide chemical diversity and concentrations of metabolites 13; these approaches can be applied to the study of retinal vascular diseases 14. Han et al. screened the metabolomic profiles of aqueous humor in wet age-related macular degeneration (wAMD) patients 15, and another study reported the identification of novel plasma biomarkers of wAMD 16. *Paris* et al*.* observed metabolic dysregulation in ischemic retinopathy and suggested that the overactivity of the arginine-to-proline pathway might be considered a therapeutic target in treating diabetic retinopathy (DR) 17.

Metabolomics analysis has also been applied in ROP in human samples 18 and animal models 19. In our previous study, we revealed significantly altered plasma metabolites in ROP patients who required treatment by performing untargeted metabolomics, in which a large proportion are amino acids and derivatives were identified 10. We herein performed targeted metabolomics analysis with a specific kit comprising 31 kinds of amino acids and derivatives (Table 2). The levels of 4 metabolites (citrulline, creatinine, arginine, and aminoadipic acid) were significantly different in the plasma of ROP infants compared with the controls. In particular, citrulline has also been identified to be increased in the plasma of ROP patients in the screening study of untargeted metabolomics 10, and this study confirmed its potential as a diagnostic biomarker for the screening of ROP. Although the AUC values of these altered metabolites did not indicate that they would be ideal biomarkers independently, the AUC was increased to 0.8703 when a panel of all 4 metabolites was used according to logistic regression analysis, indicating the potential of metabolomics of amino acids and derivatives in ROP diagnosis. Moreover, we identified significant correlations between the ROP stage and plasma levels of citrulline, creatinine, and aminoadipic acid (Fig. 3).

According to the KEGG analysis shown in Table 4, arginine biosynthesis and arginine and proline metabolism are the pathways enriched by the altered metabolites, and arginine is a key amino acid that participates in all of the involved pathways.

The plasma levels of arginine and citrulline have been shown to be elevated in patients with DR, and the pathways related to arginine and citrulline have been recognized to be dysregulated in DR 20. In the present study, increased levels of arginine and citrulline were also identified in the plasma of ROP patients. A recent study indicated that long-term use of L-citrulline is effective in the prevention of retinal vascular dysfunction induced by diabetes mellitus, and it may have a potential prophylactic effect in DR 21. Nitric oxide synthase (NOS) could oxidize arginine to citrulline and induce the production of nitric oxide (NO), and diabetes upregulated NO levels in retinal pigment epithelium (RPE), indicating that NO may alter the function of RPE leading to DR pathogenesis 22. As a regulating enzyme of arginine metabolism, arginase 2 participates in neurodegeneration during ROP pathogenesis, and deficiency of arginase 2 ameliorates the survival and function of neurons and reduces retinal neurodegeneration *in vivo* 23, 24. In addition, the level of creatinine was downregulated and the level of aminoadipic acid was upregulated in the plasma of ROP, and the roles and involved mechanisms of creatinine and aminoadipic acid in retinal neovascular diseases are worth a deep investigation.

Previous proteomic analysis studies revealed numerous plasma proteins that were differentially expressed in patients with ROP, such as complement C3 component, fibrinogen, and mitochondrial 25, 26. Further functional studies are needed to investigate whether these significantly altered proteins participate in metabolic regulations.

In this study, the following limitations should be taken into consideration: (1) indexes such as gestational age, birth weight, and postmenstrual age at blood draw were different between the ROP group and the control group, and the results may be influenced by these differences; (2) there was a relatively small number of subjects, especially in the control group (25 infants); and (3) among premature infants, many have ROP but do not require treatment, and such an intermediate group (stage 1) was not included in the present study. Thus, the dysregulated metabolites identified in this study should be validated with a larger population of ROP patients and controls in future studies.

In conclusion, this metabolomics analysis targeting amino acids and derivatives identified higher levels of citrulline, arginine, and aminoadipic acid and lower levels of creatinine in the plasma of ROP patients, and the related metabolic pathways might be involved in the pathogenesis of ROP.

## Figures and Tables

**Table 1:** Demographic variables of the subjects in the ROP and control groups.

Variable	ROP	Control	P-value
Gestational age in weeks (mean±SD)	29.09±2.23	31.29±2.33	0.0001
Birth weight in g (mean±SD)	1223.33±394.23	1508.8±449.29	0.0044
Postmenstrual age at blood draw in weeks (mean±SD)	39.44±3.20	37.98±2.30	0.0409
Body weight at blood draw in g (mean±SD)	2880.29±940.19	2600.4±666.21	0.2503
Sex (male/female)	34/24	13/12	0.57646

**Table 2:** Screening of amino acids and derivatives between the ROP group and the control group by targeted metabolomics.

Metabolite name	Transitions	Average concentration of ROP (µM)	Average concentration of control (µM)	Fold change	p-value	FDR
citrulline	176.1/159.2	39.6513	30.7085	1.2912	0.0012	0.0372
creatinine	114.1/44.0	11.4593	14.3750	0.7972	0.0036	0.0558
arginine	175.1/70.0	77.8832	61.4484	1.2675	0.0109	0.1126
aminoadipic acid	162.0/98.0	4.3091	2.5833	1.6681	0.0258	0.2000
tyrosine	182.1/136.1	101.1252	118.0665	0.8565	0.0507	0.3143
lysine	147.1/84.0	229.0000	195.8406	1.1693	0.0815	0.3933
cystine	241.1/151.9	99.4454	116.9984	0.8500	0.0888	0.3933
tryptophan	205.1/146.3	62.3735	55.9101	1.1156	0.1185	0.4242
isoleucine	132.1/86.2	61.1096	70.0279	0.8726	0.1307	0.4242
asparagine	133.1/74.2	65.1200	71.5070	0.9107	0.1440	0.4242
hydroxyproline	132.1/86.1	92.0505	80.2571	1.1469	0.1524	0.4242
aspartate	134.0/74.1	34.8977	25.6973	1.3580	0.1642	0.4242
choline	104.1/60.0	160.7088	125.7474	1.2780	0.1931	0.4605
histidine	156.1/110.1	288.7739	202.3598	1.4270	0.2181	0.4829
glutamine	147.0/84.1	825.4253	868.8833	0.9500	0.2372	0.4902
creatine	132.1/90.0	67.7460	63.2528	1.0710	0.3064	0.5673
glycine	76.0/30.1	334.3902	366.2055	0.9131	0.3111	0.5673
methionine	150.1/133.0	49.6478	44.5628	1.1141	0.3506	0.5804
proline	116.1/70.1	201.5363	210.6346	0.9568	0.3557	0.5804
spermidine	146.2/72.2	13.2098	7.3157	1.8057	0.3768	0.5840
serine	106.0/60.0	167.9776	189.2015	0.8878	0.3985	0.5883
taurine	126.0/44.1	145.3126	239.9024	0.6057	0.4326	0.6096
alanine/sarcosine	90.1/44.0	385.9632	368.7197	1.0468	0.4743	0.6205
glutamate	148.1/84.1	162.3295	173.6118	0.9350	0.4804	0.6205
ornithine	133.1/70.1	117.3583	122.2741	0.9598	0.5054	0.6254
cysteine	122.1/59.0	3.2110	3.2456	0.9893	0.5245	0.6254
putrescine	89.0/72.0	0.3470	0.3151	1.1011	0.5940	0.6820
phenylalanine	166.1/103.1	72.5253	75.6784	0.9583	0.6607	0.7315
leucine	132.1/86.2	106.3011	108.9801	0.9754	0.6969	0.7450
valine	118.1/72.1	208.3944	198.6853	1.0489	0.7337	0.7582
threonine	120.0/74.0	258.8024	255.8157	1.0117	0.9961	0.9961

FDR, false discovery rate.

**Table 3:** Diagnostic values of citrulline, creatinine, arginine and aminoadipic acid in the plasma of the ROP infants.

Metabolomics	Cutoff value (µM)	Sensitivity (%)	Specificity (%)	AUC
citrulline	>32.04	75.86	64.00	0.7221
creatinine	<14.29	75.86	56.00	0.7000
arginine	>61.64	65.52	60.00	0.6759
aminoadipic acid	>1.135	60.34	68.00	0.6545

**Table 4:** KEGG pathway analysis of the altered metabolites in the plasma of the ROP infants (selection counts≥2).

PathwayID	Definition	Selection Counts	Metabolites
hsa01100	Metabolic pathways	4	arginine; citrulline; creatinine; aminoadipic acid
hsa01230	Biosynthesis of amino acids	3	arginine; citrulline; aminoadipic acid
hsa00330	Arginine and proline metabolism	2	arginine; creatinine
hsa00220	Arginine biosynthesis	2	arginine; citrulline
